# Evaluation and Management of Ureteric Calculi in a Secondary Care Hospital in Northeast India: An Observational Study

**DOI:** 10.7759/cureus.99678

**Published:** 2025-12-19

**Authors:** Suvam Kumar, Augustine Shitiri, Temsula Alinger

**Affiliations:** 1 General Surgery, Christian Institute of Health Sciences and Research, Chümoukedima, IND; 2 Urology, Christian Institute of Health Sciences and Research, Chümoukedima, IND

**Keywords:** extracorporeal shockwave lithotripsy, ureteric calculi, ureteric stones, ureteroscopic lithotripsy, urinary tract infections

## Abstract

Background and aim

Ureteric calculi are a common urological condition that affects a large number of individuals globally. In Nagaland, Northeast India, the incidence is increasing, likely due to the consumption of high-fat, meat-based, and spicy foods. This study aims to evaluate and compare the clinical outcomes of extracorporeal shock wave lithotripsy (ESWL) and ureteroscopic lithotripsy (URSL) in the management of ureteric stones. Specifically, it assesses therapeutic outcomes and postoperative complications associated with each modality.

Materials and methods

This observational study included 101 patients with ureteric calculi measuring 5-20 mm. Diagnosis was established using X-ray of the kidneys, ureters, and bladder (KUB), ultrasonography KUB, or CT scan. Patients were treated with ESWL (n = 20, 19.8%) or URSL (n = 81, 80.2%). Preoperative assessments included hematological and biochemical profiling and serum creatinine measurement. Postoperative complications were documented. Ethical clearance was obtained from the Institutional Ethics Committee of Christian Institute of Health Sciences and Research (ref. no. 022/202223/IEC-CIHSR; registration no. ECNEW/INST/2020/782).

Results

The majority of patients were male (n = 72, 71.3%) and aged 30-50 years (n = 63, 62.4%). Flank pain was the most common presenting symptom (n = 88, 87.1%). ESWL was performed exclusively for proximal stones (n = 20, 100%), whereas URSL was used for both distal (n = 37, 45.7%) and proximal stones (n = 44, 54.3%). Stone-free rates were 18/20 (90.0%) for ESWL and 69/81 (85.2%) for URSL (p = 0.849). Postoperative complications occurred less frequently in the ESWL group (n = 1, 5%) than in the URSL group (n = 18, 22.2%).

Conclusions

ESWL is effective for proximal stones smaller than 10 mm, whereas URSL is effective for stones up to 15 mm, regardless of location. Both ESWL and URSL achieved high stone-free rates with acceptable postoperative complications, with ESWL showing slightly fewer complications for proximal stones and URSL demonstrating effectiveness for stones at all locations.

## Introduction

Ureteric calculi are a common urological condition affecting millions worldwide, characterized by the formation of mineral deposits in the urinary tract [1]. The estimated prevalence is 2-3%, with a lifetime risk of 12% for white males and 5-6% for white females [2,3]. Given the potential for serious complications, timely and effective management is essential. The condition is often marked by recurrent episodes of severe flank pain radiating toward the groin. Acute ureterolithiasis is frequently associated with nausea and vomiting, and lower urinary tract symptoms may develop as stones approach the bladder. This distinctive clinical presentation, often accompanied by hematuria (approximately 85%), allows for a relatively straightforward presumptive diagnosis in the emergency setting. However, a definitive diagnosis requires imaging, preferably without contrast enhancement [4].

Management of ureteric calculi has evolved considerably over the past few decades, with minimally invasive techniques such as extracorporeal shock wave lithotripsy (ESWL) and ureteroscopic lithotripsy (URSL) becoming mainstays of treatment. The choice of modality depends on several factors, including stone size, location, degree of obstruction, renal function, and patient preference, as outlined in guidelines from the American Urological Association (AUA) and the European Association of Urology (EAU). Stone size, shape, location, and ureteral anatomy influence spontaneous passage. Stones smaller than 5 mm often pass without intervention, whereas stones larger than 7 mm or those remaining stationary for four to six weeks typically require surgical management [5]. The two most frequently used procedures are ureteroscopy with laser lithotripsy and stone basketing, and ESWL. ESWL is often preferred for proximal and smaller stones due to its non-invasive nature, while URSL is generally favored for mid- and distal ureteric stones because of higher clearance rates and immediate results [5-9].

Despite advances in diagnosis and treatment, regional disparities in healthcare access and procedural infrastructure continue to affect patient outcomes, particularly in under-resourced areas such as Northeast India. Secondary care hospitals in these regions often face unique challenges, including limited availability of advanced imaging, specialist care, and patient awareness. Socioeconomic and demographic factors may further influence disease presentation and progression. This study aims to evaluate the stone-free rates of ESWL and URSL and to observe the postoperative complications associated with these procedures.

## Materials and methods

Study design and setting

This was a prospective, observational, hospital-based study conducted in the departments of general surgery and urology. Consecutive patients scheduled for surgical management of ureterolithiasis were enrolled after providing written informed consent. The study was conducted in accordance with the Declaration of Helsinki and received ethical approval from the Institutional Ethics Committee of Christian Institute of Health Sciences and Research (ref. no. 022/2022-23/IEC-CIHSR; registration no. ECNEW/INST/2020/782).

Inclusion and exclusion criteria

Patients aged ≥18 years diagnosed with single or multiple, unilateral or bilateral ureteric calculi measuring >5 mm and <20 mm in maximal dimension, confirmed by imaging, were included. Patients with calculi associated with renal or ureteric congenital anomalies, calculi in transplanted kidneys, or nonfunctional kidneys were excluded.

Data collection

All patients attending the general surgery and urology OPD were screened for urinary stone disease based on suggestive history. Patients were asked about prior similar complaints and treatments received. Diagnosis was confirmed using X-ray of the kidneys, ureters, and bladder (KUB), ultrasonography KUB, or CT scan. Hematological and biochemical profiles were obtained, and serum creatinine levels were recorded to assess kidney function. Patients meeting the inclusion criteria were assigned to either URSL or ESWL. Treatment selection followed existing USI, AUA, and EAU guidelines, with patient preference considered when both options were available [8].

Treatment success was assessed in terms of stone-free status. For URSL, stone-free status was checked immediately post-procedure using X-ray KUB or non-contrast CT (NCCT) KUB. For patients undergoing ESWL, multiple sessions were performed if needed, with stone-free status assessed by X-ray post-procedure. Postoperative complications, residual calculus, pain duration, and the need for double-J (DJ) stenting were recorded.

Control of confounding

Potential confounders, including age, sex, stone size, stone location, comorbidities (e.g., diabetes, hypertension, and chronic kidney disease), and history of prior urolithiasis, were prospectively recorded. These variables were adjusted for in the analysis using multivariate logistic regression to identify independent predictors of treatment success.

Procedure selection

ESWL or URSL was chosen based on stone location, size, and patient preference, in accordance with USI, AUA, and EAU guidelines [9-11].

Sample size estimation (calculation)

The minimum sample size was calculated using Yamane’s formula (1967) [12] for finite populations:

$n = \frac{N}{1 + N e^2,}$

where n = sample size, N = target population size, and e = margin of error (0.05). Based on hospital records, 134 patients with ureteric calculi were admitted during the reference period. Substituting values:

$n = \frac{134}{1 + 134 \times 0.0025} = \frac{134}{1.335} \approx 101$

The final sample size was 101 patients.

Outcome measures

The primary outcome was stone-free status. Secondary outcomes included postoperative complications, pain duration, and the need for DJ stenting.

Statistical analysis

Data were entered into Microsoft Excel (2021; Microsoft Corporation, Redmond, WA, USA) and analyzed using IBM SPSS Statistics for Windows, Version 25.0 (Released 2017; IBM Corp., Armonk, NY, USA). Continuous variables were expressed as mean ± SD, and categorical variables as frequencies and percentages. Associations between categorical variables were assessed using the chi-square test. Multivariate logistic regression was applied to identify independent predictors of treatment success. A p-value < 0.05 was considered statistically significant.

## Results

The study included 101 patients. The mean age was 37.36 years, with a range of 17 to 65 years, indicating that most patients were young to middle-aged adults. There was a predominance of males, with 62 (61.4%) compared to 39 females (38.6%). This male-to-female ratio aligns with the generally higher incidence of ureteric calculi reported in males (Table 1).

**Table 1:** Demographic characteristics of the study population (N = 101)

Variable	Value
Total patients	101
Mean age (years)	37.36
Age range (years)	17-65
Male, n (%)	62 (61.4%)
Female, n (%)	39 (38.6%)

A male predominance was observed (68; 67.3%), and most patients were aged 30-50 years (57; 56.4%). Flank pain was the most frequent presenting symptom (79; 78.2%). Most URSL patients presented within one month of symptom onset, whereas ESWL patients commonly presented between one and six months. Hypertension was the most common comorbidity in both groups. Baseline hemoglobin and total leukocyte count (TLC) were comparable; however, serum creatinine was elevated in 21 (20.8%) patients.

Most patients had acidic urinary pH, and urine microscopy and culture were negative in the majority. Radiological findings demonstrated a significant association between stone location and type of intervention, with proximal ureteric stones more often treated with ESWL and distal ureteric stones with URSL (p = 0.029). Stone size and laterality did not differ significantly between groups. Hydroureteronephrosis (HDUN) severity also differed significantly, with moderate HDUN more common in the URSL group (p < 0.001) (Table 2).

**Table 2:** Radiological findings and interventions ESWL, extracorporeal shock wave lithotripsy; HDUN, hydroureteronephrosis; URSL, ureteroscopic lithotripsy; VUJ, vesicoureteric junction

Variable	Categories	ESWL, n (%)	URSL, n (%)	Total, n (%)	χ²	p-Value
Location	Proximal	20 (100%)	36 (44.4%)	56 (55.4%)	22.805	0.029
	Distal	0 (0%)	37 (45.7%)	37 (36.6%)		
	VUJ	0 (0%)	8 (9.9%)	8 (7.9%)		
Size (mm)	5-10	11 (55%)	57 (70.4%)	68 (67.3%)	2.206	0.332
	10-15	9 (45%)	23 (28.4%)	32 (31.7%)		
	15-20	0 (0%)	1 (1.2%)	1 (1%)		
HDUN	Mild	5 (25%)	28 (34.6%)	33 (32.7%)	40.06	<0.001
	Moderate	2 (10%)	43 (53.1%)	45 (44.6%)		
	Severe	0 (0%)	5 (6.2%)	5 (4.9%)		
Laterality	Unilateral	20 (100%)	74 (91.4%)	94 (93.1%)	1.857	0.173
	Bilateral	0 (0%)	7 (8.6%)	7 (6.9%)		

DJ stenting was required in 78 patients (77.2%), predominantly in the URSL group (96.3%; p = 0.001). Most patients experienced postoperative pain lasting one to five days (Table 3).

**Table 3:** Frequency distribution of DJ stent placement by intervention group Empty cells are represented by a single hyphen (-) to indicate data that were not applicable or not observed in that category. DJ, double-j; ESWL, extracorporeal shock wave lithotripsy; URSL, ureteroscopic lithotripsy

DJ stent	ESWL, n (%)	URSL, n (%)	Total, n (%)	χ²	p-Value
Yes	0 (0%)	78 (96.3%)	78 (77.2%)	22.852	0.001
No	20 (100%)	3 (3.7%)	23 (22.8%)	-	-

**Figure 1:**
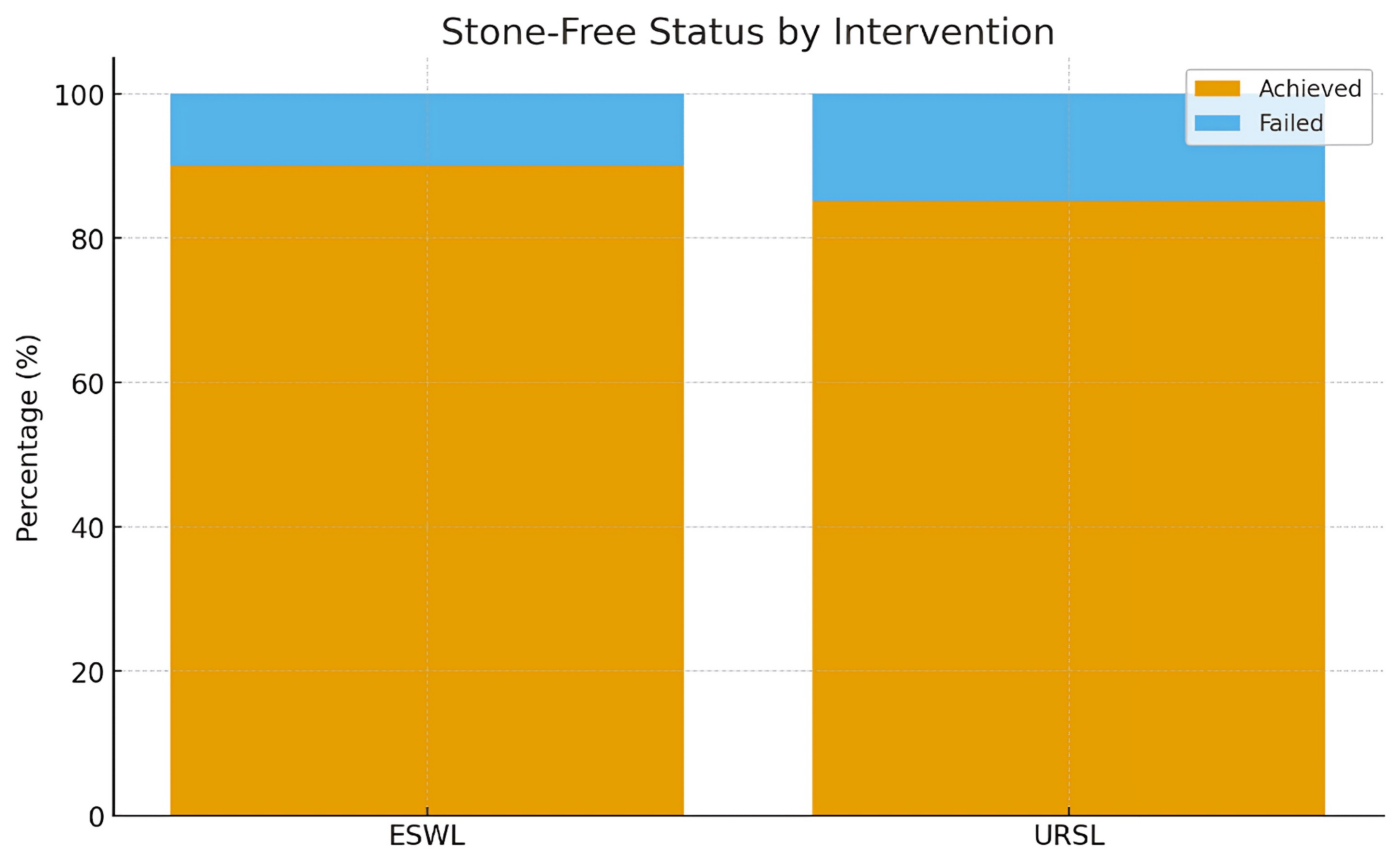
Frequency distribution of stone-free status by intervention group ESWL, extracorporeal shock wave lithotripsy; URSL, ureteroscopic lithotripsy

The stone-free rate was 90% for ESWL and 85.2% for URSL, with no statistically significant difference (p = 0.849) (Figure 1). Stone location was significantly associated with stone-free outcomes in both groups (p < 0.05) (Table 4), and stone size was significantly associated with treatment success in the URSL group (p = 0.001) (Table 5).

**Table 4:** Association between stone-free status and stone location ESWL, extracorporeal shock wave lithotripsy; HDUN, hydroureteronephrosis; URSL, ureteroscopic lithotripsy; VUJ, vesicoureteric junction

Variable	Categories	ESWL, n (%)	URSL, n (%)	Total, n (%)	χ²	p-Value
Location	Proximal	20 (100%)	36 (44.4%)	56 (55.4%)	22.805	0.029
	Distal	0 (0%)	37 (45.7%)	37 (36.6%)		
	VUJ	0 (0%)	8 (9.9%)	8 (7.9%)		
Size (mm)	5-10	11 (55%)	57 (70.4%)	68 (67.3%)	2.206	0.332
	10-15	9 (45%)	23 (28.4%)	32 (31.7%)		
	15-20	0 (0%)	1 (1.2%)	1 (1%)		
HDUN	Mild	5 (25%)	28 (34.6%)	33 (32.7%)	40.06	<0.001
	Moderate	2 (10%)	43 (53.1%)	45 (44.6%)		
	Severe	0 (0%)	5 (6.2%)	5 (4.9%)		
Laterality	Unilateral	20 (100%)	74 (91.4%)	94 (93.1%)	1.857	0.173
	Bilateral	0 (0%)	7 (8.6%)	7 (6.9%)		

**Table 5:** Association between stone-free status and stone size ESWL, extracorporeal shock wave lithotripsy; URSL, ureteroscopic lithotripsy ^*^ A p-value < 0.05 was considered statistically significant.

Group	Stone size (mm)	Stone-free status		Total	Chi-square	p-Value
		Achieved, n (%)	Failed, n (%)			
ESWL	5-10	11 (61.1%)	0 (0%)	11	2.716	0.099
	10-15	7 (38.9%)	2 (100%)	9		
	Subtotal	18 (100%)	2 (100%)	20		
URSL	5-10	54 (78.3%)	4 (33.3%)	58	13.672	0.001^*^
	10-15	15 (21.7%)	7 (58.4%)	22		
	>15	0 (0%)	1 (8.3%)	1		
	Subtotal	69 (100%)	12 (100%)	81		
Total (both groups)	5-10	65 (74.7%)	4 (28.6%)	69	15.948	0.001^*^
	10-15	22 (25.3%)	9 (64.3%)	31		
	>15	0 (0%)	1 (7.1%)	1		
	Total	87 (100%)	14 (100%)	101		

Fever was the most common complication (17; 16.9%), occurring predominantly after URSL (19.7%) (p = 0.420) (Table 6). No major complications or mortality were reported.

**Table 6:** Post-procedure complications in ESWL and URSL Empty cells are represented by a single hyphen (-) to indicate data that were not applicable or not observed in that category. ESWL, extracorporeal shock wave lithotripsy; URSL, ureteroscopic lithotripsy

Complication	ESWL, n (%)	URSL, n (%)	Total	Chi-square	p-Value
Fever	1 (5.0%)	16 (19.7%)	17	3.896	0.42
Hematuria	0 (0%)	1 (1.2%)	1	1.575	0.455
Dysuria	0 (0%)	0 (0%)	0	-	-
Burning micturition	0 (0%)	1 (1.2%)	1	1.575	0.455
Total patients with complications	1 (5.0%)	18 (22.2%)	19	1.575	0.455

## Discussion

Urolithiasis is a prevalent urologic condition with significant clinical and socioeconomic implications, affecting approximately 10-15% of the global population [6]. Surgical intervention is typically indicated for stones larger than 5 mm, symptomatic stones, or stones unresponsive to conservative treatment, as untreated calculi may result in obstructive complications and urinary tract infections [7]. According to current AUA and EAU guidelines, URSL is recommended for mid- and distal ureteric stones, while ESWL is advised for proximal stones or for patients who decline URSL [7-11].

In this study, a male predominance was noted in both treatment groups (68/101, 67.3%), consistent with previous reports suggesting higher susceptibility in males due to hormonal influences and anatomical differences in the ureter [13]. Most patients were aged 30-50 years (57/101, 56.4%), aligning with literature that identifies middle-aged adults as a high-risk group, often due to lifestyle factors such as dehydration and sedentary habits [14,15].

Flank pain was the most common presenting symptom (79/101, 78.2%), followed by dysuria and fever. Most URSL patients (53/81, 65.4%) presented within one month of symptom onset, whereas the majority of ESWL patients (12/20, 60%) presented within one to six months. A higher proportion of ESWL patients had a prior history of urological intervention (13/20, 65%), while most URSL patients were intervention-naive (63/81, 77.8%).

Hypertension was the most prevalent comorbidity (ESWL: 5/20, 25%; URSL: 22/81, 27.2%), supporting previous research that suggests an association between nephrolithiasis and high blood pressure, though the causal relationship remains complex [16,17]. Baseline laboratory parameters, including hemoglobin and TLC, were comparable between groups. Elevated serum creatinine was observed in 21/101 (20.8%), highlighting the importance of monitoring renal function in patients with urolithiasis. Urine analysis showed acidic pH, with the majority of patients exhibiting no red blood cells, no pus cells, and negative cultures (ESWL: 18/20, 90%; URSL: 75/81, 92.6%).

Radiological evaluation demonstrated a significant association between stone location and intervention type (p = 0.029). All ESWL patients had proximal ureteric stones (20/20, 100%), whereas URSL patients predominantly presented with distal ureteric stones (28/81, 34.5%). HDUN severity also differed significantly, with moderate HDUN more common in URSL patients (43/81, 53%) and mild HDUN more common in ESWL patients (5/20, 25%; p < 0.001). Most stones were unilateral (ESWL: 20/20, 100%; URSL: 74/81, 91.4%) and solitary (ESWL: 19/20, 95%; URSL: 71/81, 87.6%).

DJ stenting was required almost universally in the URSL group (78/81, 96.3%) but was not required in the ESWL group. This finding aligns with previous reports indicating that URSL often necessitates stenting, which may increase patient discomfort, cost, and follow-up requirements [18-21]. Post-procedural pain was generally mild in both groups, lasting one to five days. The routine use of DJ stents in URSL cases is clinically significant in secondary care settings, as it affects patient comfort, cost, and follow-up burden. Patient satisfaction and quality of life are important considerations in treatment selection, with prior studies reporting higher satisfaction rates for ESWL compared to URSL [22].

Both interventions achieved high stone-free rates: 90% for ESWL (18/20) and 85.2% for URSL (69/81), with no statistically significant difference. Stone-free status was strongly associated with stone location in the URSL group, with distal stones more likely to clear than proximal stones (p = 0.013). Stone size also influenced URSL outcomes, as stones ≤10 mm had higher success rates (54/58, 78.3%). Smaller proximal stones (<10 mm) were effectively cleared with ESWL (11/11, 100%), supporting its role as a minimally invasive option for proximal ureteric stones [23-25].

Complication rates were low in both groups. Postoperative fever occurred in one ESWL patient and 18 URSL patients, with hematuria noted in 1/18 URSL cases (1.2%). This aligns with previous literature indicating that ESWL is less invasive with fewer adverse events, whereas URSL provides faster clearance but carries a higher risk of minor complications [26,27].

Overall, urolithiasis is a common condition with a global prevalence of 10-15%. Stones >5 mm typically require intervention to prevent complications such as obstructive anuria or urinary tract infection. According to AUA and EAU guidelines, ESWL and URSL remain safe and effective treatment options for appropriately selected patients.

In this study, ESWL was primarily applied to proximal stones and did not require routine DJ stenting, minimizing patient discomfort and procedural cost. URSL, primarily performed for distal stones, required DJ stenting in 96.3% of cases, affecting patient comfort but ensuring procedural efficacy. Both modalities demonstrated high stone-free rates, with ESWL showing slightly fewer complications (5%) compared to URSL (22.2%), which were mainly transient fever and minor urinary symptoms. The highest likelihood of stone clearance was observed in solitary stones measuring 5-10 mm in both groups.

These findings support existing literature indicating that ESWL is less invasive with fewer complications, whereas URSL provides faster clearance, particularly for larger or distal stones. Both modalities are safe and effective and can be successfully applied in resource-limited secondary care hospitals.

Limitations

This single-center observational study, with a relatively small sample size and no control group, limits the generalizability of its findings. Alternative interventions, such as percutaneous nephrolithotomy and ureterolithotomy, were not evaluated. Stone-free status was assessed using X-ray KUB rather than NCCT, which may have led to underdetection of radiolucent stones. The absence of long-term follow-up represents another important limitation [28]. Further multicenter studies incorporating broader treatment modalities for ureteric calculi are needed to provide a more comprehensive understanding of outcomes. Additionally, improving healthcare infrastructure and securing funding for comprehensive research in Northeast India are essential to enhance the diagnosis and management of ureteric calculi. Secondary outcomes, including economic considerations and quality of life, should also be included in future studies.

## Conclusions

ESWL is highly effective for proximal ureteral stones smaller than 10 mm, offering a minimally invasive procedure with minimal complications. URSL can be performed for stones up to 15 mm regardless of location; however, it requires DJ stenting and carries a higher risk of postoperative complications.

Patient-specific factors, such as stone characteristics, comorbidities, patient preferences, and institutional capabilities, should guide clinical decision-making. Both interventions are suitable for secondary care hospitals, providing safe and effective treatment of ureteric calculi in Northeast India. Future multicenter studies with larger cohorts, a wider range of treatment options, and extended follow-up are recommended to further refine treatment strategies and improve patient outcomes.
